# Health support of people with intellectual disability and the crucial role of support workers

**DOI:** 10.1186/s12913-023-10206-2

**Published:** 2024-01-02

**Authors:** Kim Nijhof, Fleur H. Boot, Jenneken Naaldenberg, Geraline L. Leusink, Kirsten E. Bevelander

**Affiliations:** 1 Department of Primary and Community Care, Radboud University Medical Center, Nijmegen, The Netherlands; 2 Academic collaborative Intellectual Disability and Health - Sterker op Eigen Benen (SOEB), Nijmegen, The Netherlands

**Keywords:** Intellectual disability, Support workers, Health and well-being, Healthcare needs, Training needs

## Abstract

**Background:**

People with intellectual disability have a poorer health status than the general population. In The Netherlands, support workers play a key role in meeting health support needs of people with intellectual disability. Research on how people with intellectual disability and their support workers experience the support worker’s role in preventing, identifying, and following up health needs of people with intellectual disability is scarce. To enhance health support of people with intellectual disability it is crucial that we understand how health support is delivered in everyday practice. Therefore, this study investigated experiences of people with intellectual disability and support workers with the health support of people with intellectual disability.

**Method:**

Data collection consisted of six focus group (FG) discussions with between four and six participants (*N* = 27). The FGs consisted of three groups with support workers (*n* = 15), two groups with participants with mild to moderate intellectual disability (*n* = 8), and one group with family members as proxy informants who represented their relative with severe to profound intellectual disability (*n* = 4). The data was analysed thematically on aspects relating to health support.

**Results:**

We identified three main themes relevant to the health support of people with intellectual disability: 1) dependence on health support, 2) communication practices in health support, and 3) organizational context of health support. Dependence on health support adresses the way in which support workers meet a need that people with intellectual disability cannot meet themselves, and communication practices and organizational context are identified as systems in which health support takes place.

**Conclusion:**

This study investigated experiences with the health support of people with intellectual disability from the perspectives of people with intellectual disability and support workers. We discuss the dependence of people with intellectual disability and the complexity of health support in everyday practice. We provide practical implications that can strengthen support workers in the provision of health support for people with intellectual disability in everyday practice. The findings of this study emphasize the need for intellectual disability care-provider organizations to establish policies around consistency in support staff to make it easier to identify and follow up health needs, and an environment where support staff can develop their expertise concerning communication practices, lifestyle choices, and identifying and following up health needs.

**Supplementary Information:**

The online version contains supplementary material available at 10.1186/s12913-023-10206-2.

## Background

People with intellectual disability have a poorer health status compared with the general population [1, 2]. They have higher (chronic) morbidity and mortality rates [3], including premature death from preventable causes [4, 5]. Avoidable differences in health status are related to personal, institutional, or systemic drivers [6, 7]. People with intellectual disability often depend on support persons such as caregivers (e.g., a family member or guardian) or support workers (e.g., employees with direct client contact and/or intermediaries between people with intellectual disability and health professionals) [7, 8]. Research on experiences with support worker’s role in preventing, identifying, and following up health needs of people with intellectual disability is scarce. Therefore, the aim of this study is to investigate health support in everyday practice through the experiences of people with intellectual disability and support workers.

Having an intellectual disability is characterized by significant limitations in both intellectual functioning and adaptive behaviour as expressed in conceptual, social, and practical skills [9]. An intellectual disability originates before adulthood and manifests as a limited ability to meet the standards of personal independence in domains such as communication, self-care, or self-direction [5]. These characteristics translate into low health literacy and health-related challenges, such as limited awareness of being ill or reasoning about the nature, causes, and consequences of diseases and difficulty expressing pain or discomfort [10]. In The Netherlands, the majority of people with intellectual disability have supported living arrangements, either independently, with family or in group homes, and are cared for by support workers who play a key role in ensuring that the health needs of people with intellectual disability are being met [11]. In a stakeholder analysis of health promotion for people with intellectual disability, support workers at group homes and day-activity facilities were ranked as most influential and important to facilitate successful health promotion for people with intellectual disability, because of role modelling, having a signalling function, and enabling collaboration with caregivers and other stakeholders [12]. Support workers can help to 1) prevent, 2) identify, and 3) follow up health problems [13, 14].

Supporting people with intellectual disability in prevention of health problems leads to healthier lifestyle choices and healthier behaviour [15, 16]. Health problems can be avoided by preventing a disease from occurring, for example by adopting a healthy lifestyle, reducing sedentary behaviour, proper sleep habits, and hygiene [17–19]. Other forms of prevention concern preventive health assessments and recommended age- and gender-specific screening to facilitate timely intervention [10]. People with intellectual disability may understand what healthy behaviour is, but they often need support to integrate healthy behaviour into their daily routine [15, 16]. However, most support workers are not equipped to support healthy behaviour, nor are they educated to identify health problems [20].

Symptoms of health needs are often poorly recognized or understood by the support persons of people with intellectual disability [21], especially when they lack the awareness, knowledge, and skills to accurately assess signs of health problems and understand health needs [22]. In addition, many people with intellectual disability present atypical symptoms of health problems or cannot identify or communicate their health needs [23–25]. Physical as well as mental health issues or discomfort are expressed through changes in behaviour such as self-injuries, lack of appetite, or sleeping problems [26]**.** Therefore, identifying health problems of people with intellectual disability is crucial for timely intervention [13, 27].

Following up health needs helps to manage and minimize the impact of existing health problems and stop or delay their progression [28–30]. For support workers, this includes supporting clients to access health services and to adhere to recommendations made by health professionals [31]. Support workers are key in communication between a person with intellectual disability and the health professional [13]. They can inform the health professional on relevant personal or background information [32] and support people with intellectual disability to obtain, process, and understand health information or to implement the medical advice provided by the health professional [33].

Support in preventing, identifying, and following up on health problems of people with intellectual disability is crucial to improve the health status of people with intellectual disability. Traditionally, caregiving focussed on basic daily living activities [34]. Nowadays however, support workers are expected to take responsibility for their client’s health [35]. Yet, support workers often do not have a health-related or medical background [20]. Research that aims to improve the health and well-being of people with intellectual disability often recommends the education and training of support workers on the health and healthcare needs of people with intellectual disability as an important way forward [14, 22, 36–40].

In light of the considerable evidence on the crucial role of support workers in the health and healthcare needs of people with intellectual disability, it is important to learn more about how people with intellectual disability and support workers experience the provision of health support in everyday practice. In this paper, health support is defined as health support in preventing, identifying and following up health problems of people with intellectual disability provided by support workers in everyday settings. By investigating the health support needs of people with intellectual disability, we create a better understanding of the role of support workers and can enhance appropriate and attainable health support in everyday practices. Therefore, this study addressed the following research question: *‘What are the experiences of people with intellectual disability and support workers with the health support of people with intellectual disability?’.*

## Methods

### Study design

This study adopted a qualitative design with focus group (FG) discussions, which provide the opportunity for discussions and facilitate interaction [41]. Through these FGs, which took place between November 2021 and March 2022, we enabled participants to share experiences and perspectives that contributed to the understanding of the health support of people with intellectual disability. This study was submitted to the Ethical Review board of Radboud University Medical Center (Registration number 2021–7540), which waived the need for a full review according to the Dutch Medical Research with Human Subjects Law (Wet Medisch-wetenschappelijk Onderzoek met mensen (WMO)). We followed the ethical principles of the Declaration of Helsinki and GPDR regulations.

### Participants

We held FGs with support workers providing health support and receivers of health support. Support workers worked at living arrangements, day-activity facilities, or they provided ambulatory support to people with intellectual disability. Receivers of health support included adults with intellectual disability, and family members of people with severe or profound intellectual disability who were closely involved in health care and could serve as proxy informants. Including proxy informants is a common way to gather information from people with intellectual disability who cannot express themselves and has been used in other studies [42–44]. Participants were recruited through 1) the network of the academic collaborative ‘Stronger on your own feet’, a collaboration between the Radboud University Medical Center and care-provider organizations for people with intellectual disability in The Netherlands with a range of 80–800 locations, delivering care to 2500–9000 people with intellectual disability, and 2) a Dutch association for professionals in social work with over 5000 members that work at care-provider organizations throughout The Netherlands. The gatekeepers of these organizations (managers and support workers) were informed about the study and asked to help recruit participants. All participants received the study’s information leaflet. The information leaflet and consent form were adjusted for people with intellectual disability in easy-to-understand material, for example, by larger font size, fewer words per row, and visual aids such as pictures and an information video. The adjusted informed consent materials were developed by the author (KN) in collaboration with co-researcher (AvC) who has a mild intellectual disability. Potential participants could approach the first author (KN) with questions or to express interest by email, phone, or through their caregiver.

Support workers were included when they were working professionally with people with intellectual disability at supported living arrangements, day-activity facilities, or ambulatory support. People with mild to moderate intellectual disability were included in this study if they were over 18 years old, received care from a care-provider organization for people with intellectual disability, were capable of giving informed consent, and were able to express their experiences and perspectives regarding health. People with severe to profound intellectual disability were represented by proxy informants (family members). Proxy informants were included if they were over 18 years old and actively involved in taking care of their relative with severe to profound intellectual disability. Proxy informants were instructed to speak from the perspective of their relative with intellectual disability. We held six FGs, the sixth of which did not uncover new information and therefore data saturation was achieved. An overview of participants per FG is shown in Table 1.

**Table 1:** Overview of FG participants

Focus group	Participant group (*n* = 27)	Gender F/M	Mean age (SD)	Location FG
1 (pilot)	Support workers (*n* = 4)	4/0	59 (5.07)	Face to face
2	Support workers (*n* = 4)	4/0	48 (6.48)	Online
3	Support workers (*n* = 6)	6/0	40 (6.22)	Online
4	People with mild to moderate intellectual disability (*n* = 5)	4/1	52 (17.92)	Face to face
5	People with mild to moderate intellectual disability (*n* = 4)	3/1	27 (3.86)	Face to face
6	Proxy informats of people with severe to profound intellectual disability (*n* = 4)	3/1	53 (8.26)	Face to face

### Study procedures

The semi-structured FG guide (see Additional file 1) was developed by the authors (KN, FB, KB, JN, GL) based on literature as described in the introduction and supplemented by their expert opinion and the co-researcher’s (AvC) expertise by experience. The authors are experts in the field of organizational sciences (KN), intellectual disability medicine (FB, GL), health promotion, communication and citizen science (KB), health inequity in context (JN), and general practice (GL). The FG guide included five topics: 1) general health of people with intellectual disability, 2) prevention of health problems, 3) identifying health problems, 4) following up on health problems, and 5) support worker’s training needs in health support. The pilot testing of the FG and its evaluation resulted in the addition of an elicitation technique (brainstorming) at the start of each FG to encourage participants to interact. Data from the pilot were included because of the rich information obtained from the participating support workers.

All FGs were moderated by the first author (KN) and accompanied by either a co-author (FB) or research assistant who took fieldnotes. After an introduction round, the moderator (KN) started the brainstorming on the general health of people with intellectual disability. Building from that, the moderator guided the FG to discuss the remaining four topics. The FGs’ duration was approximately 60 min with people with intellectual disability and 90 min with support workers or family members. All FGs were audio-recorded after obtaining permission from the participants, transcribed intelligent verbatim, and pseudonymized before analysis.

### Data analysis

The data were analysed thematically, supported by ATLAS.ti 9.1.6 software [45]. Data analysis consisted of several steps, as described by Braun and Clarke, 2006 [46].

First, the first author (KN) read the transcripts to familiarize with the data and relevant sections of the transcripts, followed by open coding, selecting fragments relating to everyday support for the health and healthcare needs of people with intellectual disability. Our objective was to collect experiences with health support from the perspectives of receivers and providers, rather than comparing data retrieved from the different participant groups. Therefore, we coded all data as a unified dataset. Second, the quotes and the codes were regularly discussed by the authors (KN, FB, KB, JN), and additionally with the co-researcher (AvC). This resulted in a conceptual coding structure that was systematically applied to three transcripts by the main author (KN) and discussed regularly between three of the co-authors (KN, FB, KB). Third, after these three transcripts were coded, KN, FB and KB discussed which codes were overlapping and could be merged. For example, ‘familiarity’ was merged with ‘knowing a person’. This resulted in the final coding scheme applied to all transcripts by the main author (KN). Fourth, the main author (KN) clustered the codes into subthemes, and discussed this with two of the co-authors (FB, KB). This resulted in 20 subthemes. For example, ‘support workers consult health professional’ and ‘support workers consult one another’ were clustered into the subtheme ‘support workers consult stakeholders’. Fifth, patterns across the subthemes were identified to further combine and/or merge subthemes under themes. This step resulted in seven themes representing different topics discussed as relevant to health support—for example, ‘support nutrition’ as a subtheme under the theme ‘lifestyle choices’. During the sixth and last step of the analysis process, we (KN, FB, KB) re-evaluated and (re)defined names of the (sub)themes. We categorized the subthemes under three main themes to provide a conceptual framework of the health support of people with intellectual disability.

## Results

**Fig. 1:**
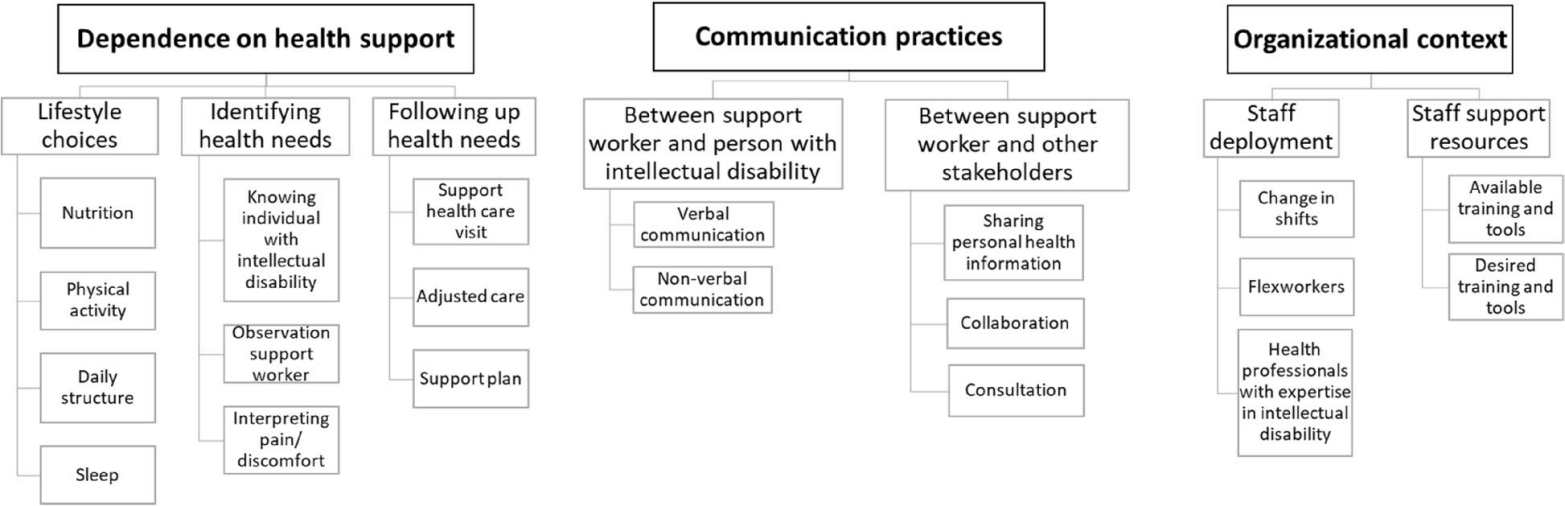
Conceptual framework of themes and subthemes regarding health support of people with intellectual disability

Data analysis resulted in a conceptual framework of health support of people with intellectual disability as shown in Fig. 1. The three main themes of the conceptual framework provide insights beyond the topics of the FG guide. The main theme, dependence on health support, relates to the FG guide topics prevention, identifying, and following up, and therefore directly to the practical provision of health support. The other two main themes—communication practices and organizational context- refer to the systems in which health support takes place. Illustrating quotes are translated from Dutch to English.

### Dependence on health support

Participants discussed health support in terms of dependence on health support in lifestyle choices, identifying health needs, and following up on health needs.

#### Lifestyle choices

Adopting a healthy lifestyle and making healthy choices were considered a challenge or sometimes even stressful for people with intellectual disability, because they experience difficulty in overseeing the consequences of (un)healthy choices and behaviour. They depended on others to make choices, such as support worker’s choices about nutrition, physical activity, daily structure, and sleep. Support workers struggled to find a balance between dependence and giving autonomy regarding healthy choices. The following quote illustrates this dilemma.

I saw that he had a low blood sugar level and I had to do something about it. But the thing is that they give him [person with intellectual disability with diabetes] a lot of autonomy concerning what to eat or not. This causes him a lot of tension, and it [diabetes] fluctuates. It influences the balance I think. [Support worker 6].

People with intellectual disability also struggled with their dependence on choices made by support workers. For example, when they would have liked to make different decisions than support workers made for them or when they felt influenced to make other choices.

It was very difficult. She stuffed me with food. And I told her I’m not hungry. I think because of my medication. But she replied: No you’re going to eat, it’s good for your health. [Person with intellectual disability 2].

Support workers were aware that their personal attitudes and lifestyle choices influence their support towards people with intellectual disability. For example, support workers have a serious impact on decisions about the timing and the nutritional content of food for people with intellectual disability. Although encouraging healthy behaviour was seen as part of the support workers’ role, this was not always reflected in practice. In the following quote, a person with intellectual disability wanted to adopt a healthier lifestyle, but did not feel encouraged because support workers kept providing cookies.

At our location, I can get a cookie as early as the coffee break at 10.30 a.m. Well, that is encouraging… [sarcastic]. I rarely take a cookie. And in the afternoon at 4 p.m. we have another coffee moment with a cookie. Then I think: let them encourage [a healthy lifestyle] a little more. [Person with intellectual disability 7].

Having a clear day-night rhythm and day structure was perceived as important for the health of people with intellectual disability. However, falling asleep and sleep deprivation were often problematic and affected their daily functioning and behaviour. In addition, a lack of day structure lead to restlessness during the day and sleeping problems. Support workers helped them to structure their day by, for example, creating a daily or weekly programme:

When I started living independently, my support workers created sort of a programme with me: okay, this is your daily structure and at that time the support workers come and help you with challenging stuff. And because I had not had that for a long time, I noticed what I had missed. [Person with intellectual disability 5].

#### Identifying health needs

Participants in this study experienced dependence on health support when people with intellectual disability had limited awareness to identify their own health symptoms and/or are unable to communicate about their health needs. Knowing the person with intellectual disability, observing behaviour, and interpreting signs of pain or discomfort were important factors for identifying health needs.

Knowing a person’s typical behaviour made it easier to notice atypical behaviour that could indicate health problems. This was especially important when contact with health professionals was required. Health professionals who do not know the person with intellectual disability have difficulty reading and interpreting symptoms, thereby potentially delaying the diagnosis of health problems. For example, when support workers consulted a health professional with their client, they felt they were not heard nor taken seriously by the health professional. This was related to the discomforting way health professionals approached individuals with intellectual disability, which caused stress to the person and regularly led to unfinished medical examinations. Family members and support workers emphasized the importance of medical professionals listening to help them identify the health needs of a person with intellectual disability. For example, an atypical manner of expressing pain is reflected in the following two quotes:

Then I indicate that his ear smells or something like that and then they say: you have to press behind his ear to see whether he is in pain. ‘Well no, he is not in pain, so this means he does not have an ear infection.’ Then I often have to tell them: ‘well, I have known him for a long time and I just know he has an ear infection.’ He gives no indication of pain, but it is not certain that, when he does not indicate pain, his ear is not infected. [Support worker 8].

She put her hand on a hot radiator, and then at a certain point, we noticed there was something odd about the way she walked. Turned out it was totally red! She had to go to the burn centre, but she did not show any signs of pain. That [not communicating about pain] could be something symptomatic for these kinds of people. [Family member 2].

Although support workers indicated that they could recognize atypical behaviour or signs of pain or discomfort in persons who have difficulty expressing themselves verbally, they frequently struggled to figure out what could be wrong and whether its origin is physical or mental. For instance, a person with intellectual disability communicated about a stomachache, but after a while support workers suspected that this could have a mental cause, as a result of a new support worker who was causing tension in this person. Such cases require support workers to have a lot of assessment skills how to interpret expressions of pain or discomfort, and support workers have indicated their wish to be trained on identifying pain and interpreting challenging behaviour.

#### Following up health needs

Participants mentioned experiences with actions or events that took place after a health problem is identified when people with intellectual disability may need help regarding the following up of health needs. This includes acting on symptoms, adjusting care to the cognitive and physical capabilities of the person with intellectual disability, and working with a support plan to adhere to the advice of a health professional and health-related goals.

People with intellectual disability were supported in accessing healthcare services and during consultations with health professionals. This included, for example, support in making an appointment for a consultation, communicating the health problem during the consultation, and assisting in obtaining, processing, and understanding health information. During visits or afterwards, support workers checked whether a client has understood what the health professional said:

Can you repeat what the general practitioner said? Or what do you remember? Well, often that is not very much. So, on that part, they simply need support. [Support worker 7].

It was not always possible for health professionals to carry out standardized health assessments on people with intellectual disability. Participants indicated that support workers and family members can help health professionals to adjust assessments by finding creative alternatives because they know what a person with intellectual disability is capable of.

Well, the client was not feeling well, so we approached the general practitioner. The client had to pee in a cup. Of course, we did not manage to do that. Then I think: how unfamiliar are you with the client for asking him to do this? So, I got creative and put an arrow-shaped item in the toilet bowl and used that to collect his urine. [Support worker 8].

Participants with intellectual disability often had an individual support plan at their care-provider organization containing their future goals and agreements concerning day-to-day activities and guiding support workers. Once a health problem was identified, support workers recorded this in the individual support plan of the person with intellectual disability at the care-provider organization. Additionally, support workers used the support plan to follow up on health professionals’ advice concerning health problems and health-related goals. Support plans were especially helpful for temporary on-call workers who do not know a client’s habits and agreements.

When you receive care from [care-provider organization’s name], you get a support plan based on your health needs and how you would like to be supported. Goalsetting [in this case relating to healthy nutrition] is also included in this support plan. [Person with intellectual disability 1].

### Communication practices in health support

Communication was a key topic in health support, overarching the dependence theme. This included the communication between support workers and people with intellectual disability, or between support workers and other stakeholders such as family members and (healthcare) professionals.

#### Communication between support workers and people with intellectual disability

Communication between support workers and people with intellectual disability occurred in both verbal and non-verbal ways. Verbal communication consisted of supporting people with intellectual disability to share their health needs, and receiving and understanding health information. For example, (educational) health information and materials are not adjusted to the cognitive level of people with intellectual disability, and support workers translate educational health information materials in a way that clients can understand and make sure that the information is provided repeatedly.

That is how important it is to adjust. A doctor often does not understand people with intellectual disability; this also applies to educational materials of course. It is not suitable for people with intellectual disability. It requires being more specific and requires more repetition. [Support worker 7].

People with intellectual disability stated that they had difficulty talking about their health problems. They were aware of the importance of informing their support worker when they were not feeling well or needed support and realized that it is not always visible to others that something is wrong. At the same time, support workers knew that people with intellectual disability have difficulty expressing their health needs or may not know how to do this. They emphasized the importance of proactively asking clients how they are feeling and ensuring that they feel safe to share their story. The following quotes illustrate this from the perspective of a person with intellectual disability and a support worker, respectively:

You are the one who must ring the bell, you are the one who has to give an indication of how you are doing. From my personal view, it is a challenge, and then I speak specifically for myself. I personally try to tell when something is wrong. Because it’s complicated, it is difficult for someone else to notice when I go passed my boundaries. [Person with intellectual disability 3].

Creating the opportunity for that story, a moment to connect, makes it more pleasant for him to get through the day. [Support worker 3].

In addition, support workers were compelled to pay attention to non-verbal communication and to be creative in figuring out their clients’ health needs. They read body language or use visual aids, such as pictures, smileys, or colours to communicate. For example, when support workers presented smileys to a client, this person could show that they are in pain by pointing at a smiley with a sad mouth:

Then we show her the smileys again: how are your knees feeling now? And we sometimes present them upside down. If she points to a smiley with the mouth and it also has an odd colour, then we assume that she is in pain and we go to a health professional. [Support worker 2].

#### Communication between support workers and other stakeholders

Participants experienced challenges concerning communication practices within the highly multidisciplinary healthcare systems around people with intellectual disability and emphasized the importance of proper communication between support workers and other stakeholders. The multidisciplinary aspects of communication were expressed through sharing personal health information about the client and through consultation and collaboration between all stakeholders.

Sharing personal health information and agreements about a person with intellectual disability was perceived as essential, but also as problematic, given the considerable number of people involved in the care and support of a person with intellectual disability—for example, when essential health information was not shared between family and support workers.

We at the living arrangement are highly dependent on who’s going with a client [to a consultation with a health professional]. Generally, we do not go with them, so when a father, mother, or brother accompanies [the client to] the consultation, then we do not always receive information. Then the client comes back and they say that the pharmacy will deliver something and we just have to figure out what we are meant to do. [Support worker 5].

Collaboration between stakeholders and consulting one another for advice was considered essential for addressing the health needs of a person with intellectual disability. When people with intellectual disability were not capable of making their own decisions, the various stakeholders involved needed to be on the same page to optimize that person’s healthcare support—for example, to coordinate steps towards healthier living, to figure out reasons for specific behaviour, or to make health-related decisions.

Eventually, you end up in conversations: Are we going to initiate the use of medication? Well, that requires a lot of dialogue. Because you do not just introduce medication like that. And there were family members who absolutely did not favour the idea of medication. Eventually, we had many good conversations, which took a while. Also with family members and the doctor. [Support worker 4].

### Organizational context of health support

The main theme, organizational context, encompasses how care-provider organizations organize their staff and how they facilitate their employees to provide health support. Support workers and family members emphasized the role of people involved in taking care of people with intellectual disability (staff deployment) and the training and tools that are available for those people (staff support resources).

#### Staff deployment

Inconsistency in the deployment of support workers—such as changes in shifts, the employment of temporary on-call workers, and health professionals who are not familiar with persons with intellectual disability—was indicated as a barrier to providing appropriate health support. Participants mentioned struggles with changes in staff, especially regarding information transfer and following up of health needs.

A lack of consistency in support staff could affect the health of people with intellectual disability and the support they received regarding health according to participants. Engaging temporary on-call workers complicated the sense of structure for, and relatedness to, people with intellectual disability. In particular, not knowing the person with intellectual disability and not knowing that person’s personalized agreements or habits are experienced as potentially harmful for the health support of people with intellectual disability:

Because she [person with intellectual disability] is restless, because of new support workers, a change in support staff, support workers who do not know about agreements. That causes restlessness, and this causes the skin-picking, which causes other issues and physical restlessness. [Support worker 8].

In the complex multidisciplinary context, it was stressed that persons are needed to coordinate information exchange and keep an overview. For example, when one professional takes responsibility to coordinate collaboration, overviews reports from multiple professionals, and takes action on prominent matters, health needs are more likely to be identified:

Who takes responsibility for what? I think that is a big search in healthcare. Then you can say: ‘Well, [colleague’s name], you have written this four times already, who is going to do something about it? Who takes responsibility for this report?’ I think regarding this, there is a lot to win within care-provider organizations. That someone takes responsibility, someone who actually reads all the reports. Then we can discover a lot more. [Support worker 3].

Care-provider organizations varied in the way in which they organize their staff. Some care-provider organizations had intellectual disability physicians and other health professionals who are specialized in communicating with, and the behaviour and health of, people with intellectual disability. Participants experienced this as being beneficial to the health of people with intellectual disability compared to health professionals who do not have this expertise. For example, when people with intellectual disability had to visit a hospital, support workers noticed how hospital staff not knowing how to interact with a person with intellectual disability affected their well-being. Support workers suggested training health professionals about intellectual disability, because they felt that hospital staff could not meet the needs of the client and, for the client’s well-being, their presence and support were required.

In the hospital they did not understand her, so we spent a lot of time being at the hospital, to support. Because they just gave her food and expected her to eat, but she did not understand what she was meant to do with the food. [Support worker 2].

#### Staff support resources

There were differences in the way in which, and in the extent to which, care-provider organizations provided health training and tools for support workers. Support workers experienced that tools to educate people with intellectual disability about health-related topics, to identify and interpret health needs, or to follow up health needs were helpful in the provision of health support. Support workers were not always provided with tools or did not know about the tools that exist. Sometimes, they used tools that they developed themselves, based on their experiences and intuition. Support workers shared experiences with tools that help them to indicate a person’s level of pain.

Then we used the Repos, which sort of is a tool for observation. This really helped us to give meaning to, and act on, a certain behaviour of that person. So yes, these kinds of tools and expertise are very important. [Support worker 12].

Care-provider organizations provide training on general medical aspects, e.g., first aid or medication, as described in the following quote.

It [training on vital signs] has to be done every two years, such as the basics of measuring temperature, saturation, blood pressure, the basics. That are things that you just have to do when you are a support worker. [Support worker 2].

Participants indicated a need for health-related training and tools, specifically for, and adjusted to people with intellectual disability. Support workers are now forced to invent creative solutions themselves. For example, they created a doll with colours to help a client indicate pain. Support workers mentioned that they would like to learn more about the following topics: challenging behaviour, (non-verbal) communication, identifying health needs, interpreting pain or discomfort, healthy ageing, and nutrition.

## Discussion

This study has examined experiences of support workers and people with intellectual disability with health support in everyday practice. We have identified three main themes that are strongly interlinked to each other: 1) dependence on health support, 2) communication practices in health support, and 3) organizational contexts of health support. This study shows that people with intellectual disability depend on support workers regarding the health support, which takes place in a complex interdependent system where communication with various stakeholders and the organization of care play a key role. In health support in everyday contexts, it is crucial that there is consistency in support staff and that they have expertise on topics such as motivating their clients to make healthy choices, how to identify and follow up health needs, and interpersonal communication.

In line with previous research, our study shows that support workers play a crucial role in health support of people with intellectual disability [13, 27, 47]. Specifically, our study shows that people with intellectual disability depend on health support in everyday practice regarding 1) lifestyle choices and 2) identifying and following up health needs. Support workers and people with intellectual disability struggle with the extent to which people with intellectual disability can make independent and autonomous lifestyle choices. People with intellectual disability indicate especially how they depend on support in lifestyle-related decisions, while also desiring autonomy in their everyday routines. Similarly, support workers aim to promote autonomy but feel compelled to intervene when they observe their client’s stress or unhealthy choices. This illustrates the paradox whereby people with intellectual disability often depend on support to make independent decisions [48, 49]. The extent to which they are capable of making independent decisions depends on their cognitive level and is therefore different for every individual with intellectual disability [50, 51]. Support workers can promote independent decision making by, for example, asking questions or showing pictures to clarify desires, explaining options, and discussing potential consequences of decisions. In previous studies, support workers stated that this was challenging and that they needed additional knowledge and communication skills to motivate and stimulate their clients to make healthy choices, for example about nutrition and physical activity [52, 53]. Therefore, it is important that support workers are facilitated with opportunities to develop knowledge and skills to support in lifestyle-related factors in everyday practice. Care-provider organizations can provide sufficient resources for support workers to support in health promotion, embed healthy lifestyle in policies, and promote regular health checks and offer their employees opportunities to develop awareness, knowledge and skills about connecting healthy lifestyles to daily routines [54].

Besides the crucial role of support workers in lifestyle choices, our study shows their role in identifying and following up health needs. There are only a few studies that cover identifying or following up health needs in the disability sector. These studies concentrate on healthcare environments and recommend health professionals to be trained on improving communication with, and specific healthcare needs of, people with intellectual disability [55–57] In everyday practice, support workers and people with intellectual disability experience that knowing each other well helps identify health needs, because support workers recognize changes in behaviour and people with intellectual disability share their health needs more easily with someone they know. Familiarity also helps in following up health needs, because support workers know what a person is capable of (e.g., regarding understanding health information and health assessment). Our findings on the importance of familiarity between support workers and people with intellectual disability to identify and follow up health needs are in line with previous studies showing that this leads to improved health outcomes [13, 27]. Moreover, inconsistency in staff (e.g., temporary on-call workers) or unfamiliarity (e.g., between people with intellectual disability and health professionals) can lead to problematic health situations, because health problems remain unnoticed and worsen when people with intellectual disability feel unsafe to share their health needs [7, 58, 59]. Consistency in support staff is important in this, however, scarcity in support staff is a challenge for many care-provider organizations [60]. While digital technology, such as socially assistive robots, have shown promise in the assistance and relief of healthcare staff [61–64], future studies are needed to investigate how and whether these technologies can also help support workers in the everyday health support of people with intellectual disability. Additionally, clarifying expectations and who needs which information helps following up health needs. Preparing a consultation and having background information available, investigating the opportunity to book extra time for an appointment and ensuring to leave the consultation with enough information to follow up the advice given by the health professional is in line with this finding [33]. This requires intellectual disability care-provider organizations to embed these tasks to the role of support workers and enable them with time and human resources to act accordingly.

Further, our study shows that support workers communicate with people experiencing limitations in communication skills, within complex systems and with multiple stakeholders. The complexity of (non-verbal) communication concerning health needs entails support workers having communication skills at two levels: 1) the individual with intellectual disability, for example by pro-actively asking a client about well-being or observing behaviour to identify health needs and 2) the network around a client, for example, by exchanging health information with the individual with intellectual disability, family members, colleagues, and external (health) professionals. Although the complex and multidisciplinary systems in which the health support of people with intellectual disability takes place can be influenced only minimally [12], communication practices can be improved. In our study, support workers expressed their desire to learn more about (non)verbal communication with people with intellectual disability, as well as the wish that health professionals should be trained on communication with people with intellectual disability. Several studies have confirmed the importance of communication around the health of people with intellectual disability and suggest training professionals. These topics are also reflected on in literature; several studies have suggested teaching communication skills to support workers to improve health information exchange [59, 65–67], and other studies have suggested training for health professionals on communication with people with intellectual disability specifically [68–70]. Therefore, care provider organisations can facilitate communication training and make training tools accessible for their staff [71].

Intellectual disability care-provider organizations can influence expertise development in health support [12] and should take the practical challenges faced by support workers into account and incorporate them into their policies. Some care-provider organizations facilitate training to optimize health support; however, a large proportion of such training programmes are curative and often not evaluated [72]. An evidence-based learning environment can empower support staff to provide better health support to people with intellectual disability and help them to live healthier [73]. This study shows the relevance of developing support staff’s expertise in health support. Accordingly, we emphasize the need for intellectual disability care-provider organizations to establish an environment in which there is 1) a learning environment where support staff can develop their expertise concerning lifestyle choices and identifying and following up health needs and communication practices and 2) as much consistency in support staff as possible (e.g., by technology assistance) to make it easier to identify and follow up health needs.

### Strengths and limitations

A major strength of this study is that we have included the perspectives of both support workers and people with intellectual disability on health support in everyday practice. Their practical experiences have helped us understand how health support in daily setting is provided and received. However, there are also a few limitations to this study.

The data for this study were collected in the winter of 2021/2022, during which Covid-19-related restrictions were in place in The Netherlands. Therefore, the fact that two FGs with support workers were conducted online may have affected sensing non-verbal communication. The online FGs did, however, allow support workers from different organizations to join in, thereby enriching our data; this would otherwise not have been possible at a physical location because of travel distances.

We conducted separate FGs for support workers, people with mild to moderate intellectual disability and proxy informants of people with severe to profound intellectual disability. Despite the challenges to include people with intellectual disability in research, investigating their experiences and perspectives is increasingly acknowledged as being valuable [74, 75]. There are different ways to include the experiences and perspectives of people with severe to profound intellectual disability, for example, by using visual aids, translation by a (professional) caregiver or the use of proxy informants [76]. Given that none of the options have been shown to increase study validity, we avoided burdening people with severe to profound intellectual disability and included proxy informants in this study. They shared experiences from their relative with severe to profound intellectual disability, whose experiences could not have been included without their participation.

Furthermore, the different groups of participants were not mixed in a FG. Combining the groups of participants in one FG would have complicated data collection because of differences in understanding language and possible dependence and power relations, which could have prevented participants from speaking freely [77]. We did not aim to compare experiences in this study, but it may be interesting for future research to compare between groups how their experiences with health support overlap or differ.

Importantly, intellectual disability care and healthcare settings vary greatly between countries [78, 79]. Our study was conducted in The Netherlands, a country with a high standard of healthcare resources [80]. Care for people with intellectual disability is organized differently in, for example, low-resource countries, where the majority of people with intellectual disability live with, and are taken care of by family members [2, 81], and community workers are key actors for supporting and educating families [82]. As the organization of care relates to the way health support needs are met, conducting this study in differently resourced countries is likely to provide additional insights.

## Conclusions

This study has shown how people with intellectual disability depend on support staff for their health needs. Support in making healthy choices and identifying and following up health needs in particular are major aspects of support workers’ role in health support. The provision of health support in everyday practice is complicated by the fact that support workers fulfil their role within complex systems of communication with many stakeholders and challenging organizational contexts. Intellectual disability care-provider organizations are recommended to establish policies around consistency in support staff and an evidence-based learning environment to strengthen support workers’ role in the health support of people with intellectual disability.

### Supplementary Information

**Additional file 1: ** Focus group guide

## Data Availability

The datasets used and/or analysed during the current study are available from the corresponding author on reasonable request.
