# Supplementary material for: Health support of people with intellectual disability and the crucial role of support workers
Source: BMC Health Serv Res. 2024 Jan 2;24:4. doi: 10.1186/s12913-023-10206-2 (PMC10763292; doi:10.1186/s12913-023-10206-2)
Supplement: Supplementary file 1 — Additional file 1: Focus group guide [file 12913_2023_10206_MOESM1_ESM.docx]

| **Focus group guide** | | |
| --- | --- | --- |
| **Support workers** | **People with ID and family** | **How?** |
| **Topic 1: Health of people with ID [10/5 min]**  Aim: provide inspiration for the discussion and explore first thoughts on health of people with ID. | | |
| 1.1 What comes to mind when you think of ‘health’?  1.2 What are your experiences with the health of specifically people with ID? | 1.1 What comes to mind when you think of ‘health’?  1.2 (for family members) What are your experiences with the health needs of your relative with ID? | Brainstorm |
| **Discussion 2: Prevention [15/10 min]**  Aim: explore how support workers contribute to prevent health problems | | |
| 2.1 Can you give examples of your experiences with the prevention of health problems concerning people with ID? | 2.1 How do support workers help you/ your relative with ID to prevent health problems? | Discussion |
| In-depth:  How did that go?  What went wrong?  What was successful?  What would you like to learn regarding this topic? | In-depth:  What did you like? Why?  What didn’t you like? Why? |  |
| **Discussion 3: Identifying health needs [15/10 min]**  Aim: explore how support workers identify health needs of people with ID | | |
| 3.1 Can you share experiences of situations where you identified a health problem of someone with ID? | 3.1 Did a support worker ever notice a health problem you/ your relative with ID had? What happened? | Discussion |
| In-depth:  How did you notice this?  What did you do?  How did that go?  What went wrong?  What was successful?  What would you like to learn regarding this topic? | In-depth:  What did you like? Why?  What didn’t you like? Why? |  |
| Opportunity for break when requested | | |
| **Discussion 4: Follow up health needs [15/10 min]**  Aim: explore how support workers follow up on health problems of people with ID | | |
| 4.1 What are your experiences regarding the support of a person with ID when he/she has to visit a doctor? | 4.1 How do support workers help you/ your relative with ID when visiting a doctor? | Discussion |
| In-depth:  What did you do (before, during, and after)?  How did that go?  What went wrong?  What was successful?  What would you like to learn regarding this topic? | In-depth:  What did you like? Why?  What didn’t you like? Why? |  |
| **Discussion 5: Training about health of people with ID [15/0-10 min]**  Aim: explore training needs of support workers regarding health of people with ID | | |
| 5.1 If you would be offered a training programme about health of people with ID, what would you hope it includes? Which subjects should it cover (in addition to the topics we discussed)? | 5.1 What do you think support workers should learn about your health/ the health of your relative with ID? | Brainstorm |
| In-depth:  What does this training programme need in order to be successful? |  |  |
